# A Proposed Algorithm to Assess Concussion Potential in Rear-End Motor Vehicle Collisions: A Meta-Analysis

**DOI:** 10.1155/2020/9679372

**Published:** 2020-05-13

**Authors:** Manon Limousis-Gayda, Rami Hashish

**Affiliations:** National Biomechanics Institute, Los Angeles, CA 90403, USA

## Abstract

Concussions represent an increasing economic burden to society. Motor vehicle collisions (MVCs) are of the leading causes for sustaining a concussion, potentially due to high head accelerations. The change in velocity (i.e., delta-*V*) of a vehicle in a MVC is an established metric for impact severity. Accordingly, the purpose of this paper is to analyze findings from previous research to determine the relation between delta-*V* and linear head acceleration, including occupant parameters. Data was collected from previous research papers comprising both linear head acceleration and delta-*V* at the time of incident, head position of the occupant, awareness of the occupant prior to impact, as well as gender, age, height, and weight. Statistical analysis revealed the following significant power relation between delta-*V* and head acceleration: head acceleration = 0.465delta‐*V*^1.3231^ (*R*^2^ = 0.5913, *p* < 0.001). Further analysis revealed that alongside delta-*V*, the occupant's gender and head position prior to impact were significant predictors of head acceleration (*p* = 0.022 and *p* = 0.001, respectively). The strongest model developed in this paper is considered physiologically implausible as the delta-*V* corresponding to a theoretical concussion threshold of 80 g exceeds the delta-*V* associated with probability of fatality. Future research should be aimed at providing a more thorough data set of the occupant head kinematics in MVCs to help develop a stronger predictive model for the relation between delta-*V* and head linear and angular acceleration.

## 1. Introduction

Each year, over 2 million Americans sustain a traumatic brain injury (TBI) [1]. In 2010, the United States' economic burden from patients suffering from TBIs was approximately $76.5 billion [2]. The burden is not only financial, as an estimated 20% of those patients will experience long-term impairments and disabilities with symptoms including memory loss, depression, and/or cognitive difficulties resulting in large and unquantifiable human cost [3, 4]. According to the Centers for Disease Control and Prevention, the third leading cause (14%) of TBI is motor vehicle collisions (MVCs). Rear-ends are the most common type of MVCs, accounting for 40% of all accidents [5]. TBIs are graded from mild to severe based on a range of criteria experienced at the time of accident. More than 75% of the sustained TBIs are classified as mild and are also referred to as concussion [6].

Concussions are considered to potentially be the result of high linear and/or angular accelerations to the head. Research has shown that linear acceleration of the head is closely related to pressure experienced in the brain [7]. Further studies established that the increasing pressure in the brain causes neurologic dysfunction, with dysfunction levels correlated with the peak pressure experienced at the time of insult [8]. On the other hand, rapid angular acceleration of the head is responsible for the generation of shear forces resulting in high potential for brain tissue damage [9]. While there is an ongoing debate regarding the linear threshold for concussion, it has been proposed that rates of acceleration experienced by the head exceeding 80 to 90 g would result in concussion for healthy young male athletes [10, 11]. With regard to angular acceleration, concussions have been found to occur for angular head accelerations as low as 1200 rad/s^2^ [12].

In the case of rear-end MVCs, experienced accelerations at the head appear to be associated with the change in velocity of the vehicle incured during the impact (also referred to as delta-*V* or Δ*V*). Delta-*V* has traditionally been used as an indicator for the severity and magnitude of impacts and is a key predictor for occupant's injury in MVCs [13]. Although the scientific community is aware of a relation between head acceleration and delta-*V*, this relation is not quantitatively well understood. Other parameters, such as occupant position [14, 15] or body mass index [16], have also been reported to affect head acceleration for a given delta-*V*, but quantification of the effect of those parameters has yet to be conducted. The poor quantitative understanding of the aforementioned relation can be explained by the challenging nature of conducting tests to collect such data. While a number of tests have been performed using ATD (Anthropomorphic Testing Device) which were designed to simulate human response to impacts in MVCs, those come with a number of limitations. To truly assess the human response to MVCs with parameters such as awareness of impact, volunteer testing is essential. However, this task is a challenging one as these tests can only be performed in highly controlled environments to guarantee no harm to the subjects, limiting testing to subinjurious speeds.

The purpose of this meta-analysis is to determine the relation between linear head accelerations and delta-*V* in rear-end collisions, and to investigate the extent to which other occupant parameters affect this relation.

## 2. Materials and Methods

### 2.1. Search Method

An online search was performed in the following databases: Web of Science, PubMed, and Google Scholar. All databases were searched for all available years with the last search completed in June 2018. The search strategy was based on a combination of the following keywords: head acceleration, concussion, brain injury, head kinematics, delta-*V*, change in velocity, motor vehicle accident, motor vehicle collision, and rear-end. The field was further narrowed to only include published articles written in English, French, or German. Papers were appraised if they fulfilled the following criteria: studies investigating human response to impacts reporting both measured peak head acceleration and delta-*V* of the target vehicle/sled. Since vehicle manufacturers have been required to equip vehicles with headrests since 1969 and previous research has demonstrated headrest to be a limiting factor of head retraction in whiplash [17], any study not including a headrest was removed. In addition, research has shown that bracing of the occupant prior to impact by contracting his/her neck and upper torso muscles, creating stiff “springs,” may limit head movement [18]. Therefore, only studies reporting living human responses were included. It is also important to note that while both high linear and angular head accelerations have been found to be associated with concussion potential, reports on angular accelerations in the literature are very limited. Therefore, only papers examining linear accelerations were considered for this study.

### 2.2. Data Collection

The following data was collected from the selected studies:

1. Head acceleration: peak linear acceleration experienced by the head (G)
2. Delta-*V*: change in velocity at the time of impact (km/h)
3. Head position of the occupant prior to impact: neutral or out of position
4. Occupant's awareness prior to impact: yes or no
5. Gender of the occupant: male or female
6. Age of the occupant (years)
7. Height of the occupant (cm)
8. Weight of the occupant (kg)

Ultimately, 53 studies were examined, 14 of which were found to fulfil the selected inclusion and exclusion criteria, reporting a total of 139 collisions. On occasion, the collision's parameters of interest were not explicitly reported in the papers as the studies were focusing on other aspects of rear-end collisions. Namely, position of the occupant prior to impact was not reported for Anderson et al. [18], McConnell et al. [19], Scott et al. [20], Szabo et al. [21], and Tencer et al. [22]. In these instances, the initial head position was considered to be neutral unless stated otherwise by the authors. Awareness of the occupant prior to impact was not reported by Scott et al. [20] and Tencer et al. [22]; and height and weight of the occupant were not reported by Matsushita et al. [15] and Tencer et al. [22]. Multiple data imputation was performed to fill in the missing variables by averaging the outcome of three different imputation methods: the mean of the observed values for that variable, a randomly chosen value from an individual who has similar values on other variables (hot deck method), and a systematically chosen value from an individual who had similar values on other variables (cold deck method) [23].

### 2.3. Statistical Analysis

Statistical analysis was conducted with both SPSS (SPSS Inc., Chicago, IL) and R (R Project, Auckland NZ) softwares. Best fit line and multiple regression analysis were conducted with SPSS. The best fit line allowed the authors to understand the primary relation between head acceleration and delta-*V* and develop an initial model (model 1). To understand the effect of the selected parameters on the experienced head acceleration, a multiple regression was performed. Based on these findings, a quadratic model (model 2) was developed by performing another regression only including the significant predictors (*p* < 0.05). Lastly, a full linear mixed model (model 3) was fitted on those significant predictors.

## 3. Results

From the 14 studies fulfilling the selected criteria, 139 collisions were analyzed. The ascertained data set presented head accelerations ranging from 0.6 to 17.2 g and delta-*V*s ranging from 1.3 to 11.1 km/h.

**Figure 1:**
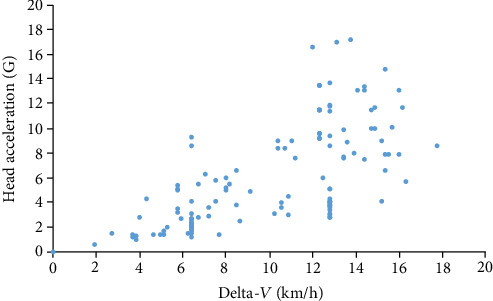
Power relationship between delta-*V* and head acceleration, regardless of gender, or occupant's head position prior to impact.

Initial curve fit analysis for the relation between delta-*V* and head acceleration indicated that the best line of fit would be a power curve (model 1) defined with the following equation (Figure 1):

(1) $$\begin{array}{c} \text{Head Acceleration}=0.465\text{Delta}‐V^{1.332}. \end{array}$$

For this model, the *R*^2^ value was found to be 0.5913 (*p* < 0.001).

Accordingly, the variable delta-*V*^2^ was developed. A multiple regression revealed that out of all the parameters, delta-*V*^2^, position of the occupant prior to impact, and gender of the occupant were the only significant predictors of head acceleration (*p* < 0.001, *p* = 0.001, and *p* = 0.022, respectively).

Consequently, a quadratic model (model 2) was developed utilizing the aforementioned significant predictors and yielded the following equation:

(2) $$\begin{array}{c} \text{Head Acceleration}=1.101+0.0962\text{Delta}‐V^{2}+2.550\;\left(\text{if out of position}\right)+1.220\;\left(\text{if female}\right). \end{array}$$

For this model, the *R*^2^ value was found to be 0.5929 (*p* < 0.001).

In an attempt to further increase the fit of the model to the data gathered, another model (model 3) was developed and yielded the following equation with a *R*^2^ value of 0.8215 (*p* < 0.001):

(3) $$\begin{array}{c} \text{Head Acceleration}=2.22045e^{-16}+0.391\text{Delta}‐V+2.138\;\left(\text{if out of position}\right)-0.576\;\left(\text{if male}\right). \end{array}$$

## 4. Discussion

The purpose of this analysis was to determine the relation between linear head accelerations and delta-*V* in rear-end collisions and to investigate the extent to which other occupant parameters affect this relation. The significant predicting parameters were found to be gender (male vs. female), and position of the occupant prior to impact (neutral position vs. out of position). Based on this information, models were developed to predict head acceleration for a given delta-*V*, accounting for gender and head position of the occupant prior to impact.

Despite the relatively low *R*^2^ values found in the first and second models, their predictions regarding head acceleration are consistent with numbers reported by the National Highway Traffic Safety Administration (NHTSA) stating that for distributed impacts, the range of delta-*V* characterizing occupants with all brain injuries is between 14.5 and 136 km/h with an average of 50.5 km/h [24]. However, it is important to note that the presented paper is addressing mild TBIs. Therefore, the threshold for this type of injury is expected to be on the lower-end of this range. In fact, Viano and Parenteau [25] used NASS data collected between 1994 and 2011 and reported that 35% of concussion occurred for delta-*V* ranging from 16 to 24 km/h and 16% of the concussions were observed for delta-*V* lower than 16 km/h. For the occupant to sustain a mild traumatic brain injury, a theoretical 80 g threshold for concussion was used based on available literature. Based on the predictions of the models (Table 1), females would experience head acceleration of 80 g for lower delta-*V* than males. Similarly, out of position occupants would experience concussion from lower delta-V when compared to in-position occupants.

While the predictions given by the first two models agree with data available from NHTSA, the predictions of the third model presented do not appear physiologically sound. This model is suggesting that head acceleration would reach 80 g for a delta-*V* higher than the delta-*V* reported in the literature corresponding to a 100% chance of sustaining a severe to fatal injury [26, 27].

Findings regarding the significant effect of gender on head acceleration are consistent with previous research reporting significantly greater and earlier peak head acceleration for females when compared to their male counterparts [28]. It can be hypothesized that gender variations in segmental geometry, body shape, and mass distributions (subcutaneous fat vs. visceral fat; waist vs. hip girth) may also play a role in occupant positioning and inertial responses to impact. On the topic, Viano [29] found the relative acceleration between head and torso to be 30% higher for females than males, and showed that the greater movements of the neck experienced by females were a result of the difference between seat stiffness and their torso's mass.

Similarly, the effect of head position prior to impact has previously been assessed and an increase in horizontal distance between the head and headrest in rear-end impacts was found to increase head velocity [30], magnitude, and timing of peak head kinematics [31]. One of the explanations lies in the fact that at the time of impact, the torso will impact the seat back and the head will be left to fall backwards as the torso is proceeding to move forward. However, research on the topic is limited as most of the research conducted to investigate neck and head kinematics for different pre-impact positions has been focused on cervical spine compression rather than acceleration experienced at the head [15].

Although statistical analysis did not find height, weight, age, and neck tension prior to impact to be significant predictors of head acceleration, the differences found between genders could be explained by the overall difference in height, weight as well as body mass distribution, and muscular differences. In addition, the adipose tissues affecting the energy absorption capacities will affect transfer mechanisms for load and kinematics between head and torso. Another parameter that was not found to be a significant predictor in this study was the awareness of the occupant and associated neck muscle tension prior to impact. Hendler et al. [32] showed that muscle tension allowed subjects to withstand higher sled acceleration without suffering significant cervical pain or whiplash injuries. In addition, Kumar et al. [33] showed a significant decrease in head acceleration for subjects expecting the impact. However, great care must be taken when reviewing papers investigating awareness effect prior to the collision as the test designs might not accurately represent the response of unprepared individuals in real-life collisions [34]. Lastly, the effect of the seat stiffness on occupant kinematics in rear-end impacts is recognized and has been found to affect head acceleration [30, 35]. In an effort to increase rear-end impact protection, Melvin and McElhaney [36] stated that in order to decrease occupant potential for injury, the seatback should provide minimal storage for elastic energy by using energy dissipating material.

It is important to note that while the theoretical threshold of 80 g was used in this manuscript, this threshold only represents the population in which the research was conducted. A majority of the available literature investigating concussion threshold has been conducted in contact sports, namely, football and boxing. The population studied in these instances is comprised of young, athletic, healthy males. Research has shown that other populations such as older adults and/or individuals with a history of migraine, prior concussion or spinal pathology are likely at a higher risk of sustaining concussion for the same experienced acceleration, and thus, likely have a lower threshold to brain injury [37–39].

Considering the presented results, it is important to address the limitations of this paper. Although the authors used their best efforts in the review and selection of the papers used for this analysis, the studies selected were heterogeneous in their study methodologies. The repeatability of these experiments is relatively low due to the myriad of difficulties associated with the complexity of controlling the aforementioned parameters. In addition, in an effort to prevent serious injury, all of the studies documenting occupant's kinematics and head responses to collision are usually tested for delta-*V* under 14.5 km/h [15, 21, 40]. It could be argued that the use of dummies and cadavers for these tests would enable the collection of data for higher delta-*V*s, but it is important to note that those options are not always able to perfectly mimic human responses to impact. Lastly, when conducting impact studies and reporting the occupant's kinematics, the current literature fails to report angular head acceleration which has been argued to be a better indicator of TBI than linear head acceleration [41].

Future research should aim to provide a comprehensive data set for head kinematics, including an analysis of head angular acceleration. Other parameters which could be potential predictors of head acceleration and concussion, such as subject awareness, neck and seat stiffness, and geometry, should also be prospectively analyzed. To obtain this comprehensive set of data, a number of options are available. First of all, reverse engineering could be performed from data available from real-life collisions [42]. Another option is to model the test performed on volunteers and using finite element model analysis in order to investigate human head response for higher delta-*V*. However, while the mathematical models and computer simulations present a real potential for accurate analysis of occupant response to impact simulations, there is a need for a comprehensive and accurate database from experimental testing conducted with a standardized methodology for optimum control of the aforementioned parameters.

## 5. Conclusions

This paper provides a first attempt at quantitatively understanding the relation between delta-*V* and head acceleration by proposing three different models. The effect of potential predictors was also investigated and revealed that gender and occupant head position prior to impact had a significant effect on the experienced head acceleration. The authors suggest that underlying factors such as height, weight, awareness of the occupant, and seat stiffness could also be contributors to the observed effects. The findings from this study may provide insight as to the factors associated with brain injury and thereby assist in the development of improved safety measures.

## Figures and Tables

**Table 1:** Predicted head acceleration based on the developed models.

Head acceleration of 80 g	Gender	Head position	Delta-*V* (km/h)
Model 1	NA	NA	47.69

Model 2	Female	In position	28.42
	Male	In position	28.64
	Female	Out of position	27.95
	Male	Out of position	28.17

Model 3	Female	In position	204.60
	Male	In position	206.08
	Female	Out of position	199.14
	Male	Out of position	200.61
